# Kidney Transplantation in Acute Kidney Injury Research: Rodent Models, Mechanisms, and Interventional Strategies

**DOI:** 10.3390/biology15211978

**Published:** 2026-11-02

**Authors:** Gang Zhang, Chen Feng, Helong Dai

**Affiliations:** Organ Transplantation Center, Xiangya Hospital, Central South University, 87 Xiangya Road, Changsha 410008, China; fan_chon@163.com

**Keywords:** kidney transplantation, acute kidney injury, ischemia–reperfusion injury, delayed graft function, rodent models, organ preservation, microvascular dysfunction

## Abstract

Kidney transplantation is the optimal treatment for end-stage kidney disease (ESKD), but transplant-associated acute kidney injury (AKI), primarily driven by ischemia–reperfusion injury (IRI), contributes to delayed graft function (DGF) and inferior long-term graft outcomes. Rodent kidney transplantation models provide a clinically relevant platform. This review summarizes the establishment and application of rat and mouse kidney transplantation models for studying transplant-associated acute kidney injury. It highlights critical variables in model design, including surgical techniques, ischemic conditions, organ preservation, donor characteristics, and immunological settings that influence experimental outcomes. It further synthesizes current mechanistic evidence on oxidative stress, microvascular injury, immune activation, regulated cell death, tissue repair and fibrosis, molecular regulatory networks, multi-omics analyses, and remote organ injury. The review also examines interventions targeting donor conditioning, organ preservation and perfusion, reperfusion inflammation, recipient therapy, and regenerative repair, while considering their experimental limitations and translational relevance. Rodent kidney transplantation models can bridge conventional renal ischemia–reperfusion models and clinical transplantation. Future progress will depend on standardized surgical procedure, clinically relevant model selection, rigorous long-term outcome assessment, and the integration of emerging molecular technologies to enhance translational relevance.

## Simple Summary

Kidney transplantation is the preferred treatment for end-stage kidney disease, but injury occurring during transplantation can affect early graft function and long-term outcomes. Rat and mouse kidney transplantation models are widely used to investigate this injury because they reproduce important steps of the transplantation process that conventional kidney injury models cannot fully capture. This review summarizes how rodent kidney transplantation models are established, the experimental variables that influence transplant-associated acute kidney injury, and the mechanisms involved, such as oxidative stress, microvascular injury, inflammation, cell death, and tissue repair. In addition, the review discusses experimental approaches aimed at reducing kidney injury during organ preservation, transplantation, and recovery, together with their current limitations. Improving model standardization, selecting experimental conditions that better reflect clinical transplantation, and integrating findings from human studies may help translate experimental discoveries into better strategies for protecting transplanted kidneys.

## 1. Introduction

Kidney transplantation is the most effective renal replacement therapy for end-stage kidney disease. However, ischemia–reperfusion injury (IRI), which is unavoidable during donor organ retrieval, preservation, and implantation, is a major contributor to early graft dysfunction and is associated with adverse long-term graft outcomes [1,2]. Acute kidney injury (AKI) induced by IRI during kidney transplantation is a central pathophysiological basis of delayed graft function (DGF). Transplant-associated AKI not only delays early graft recovery but also increases the risks of acute rejection, graft failure, and adverse long-term outcomes. As donor criteria continue to expand and marginal kidneys are used more frequently, IRI-associated AKI and DGF have become increasingly important challenges in kidney transplantation, making their mechanisms and potential interventions a major focus of investigation [3,4,5,6].

Transplant-associated AKI is not simply a consequence of ischemia. Instead, it represents a continuous pathophysiological process driven by donor factors, organ preservation, reperfusion, and host immune responses. Conventional renal pedicle-clamping models cannot fully reproduce key features of clinical kidney transplantation, such as ex vivo organ preservation, vascular reconstruction, and alloimmune activation. Rodent kidney transplantation models have therefore become an important experimental platform for studying transplant-associated AKI. Studies using these models have helped establish that transplant-associated AKI is not merely a transient consequence of ischemia but a dynamic pathological process involving tissue injury, innate immune activation, interactions with alloimmune responses, and chronic graft remodeling [7,8,9].

Rodent kidney transplantation models require substantial microsurgical expertise, and their value depends strongly on model stability and reproducibility. Rat models are widely used for microsurgical training, assessment of preservation and perfusion strategies, and donor- or recipient-directed interventions because their larger vessel diameter makes vascular anastomosis technically more manageable and reproducible [10,11,12]. Mouse models are more technically challenging, but their mature genetic tools and immunological resources provide distinct advantages for studying mechanisms of transplant-associated AKI and immune regulation [8,9,13,14]. Nevertheless, because rodents differ from humans in immune responses, renal physiology, and drug metabolism, and because these models only approximate the clinical transplantation process, experimental findings should be interpreted cautiously when considering clinical translation [7].

Previous reviews have addressed animal models and therapeutic approaches for delayed graft function [15] and emerging therapeutic strategies for ischemia–reperfusion injury in kidney transplantation [16]. The present review takes a different perspective by focusing on rodent kidney transplantation models and their applications in investigating transplant-associated AKI. It integrates model establishment, experimental conditions, mechanistic findings, and therapeutic interventions, with particular attention to the relevance and limitations of evidence derived from transplantation-based studies.

A literature search was conducted in PubMed and Web of Science Core Collection for publications available through May 2026. The search combined (“kidney transplantation” OR “renal transplantation”) AND (“acute kidney injury” OR “ischemia–reperfusion injury” OR “delayed graft function”) AND (“rat” OR “mouse” OR “rodent”), with additional searches using terms related to model establishment, surgical techniques, organ preservation, and therapeutic interventions. Primary in vivo rat or mouse kidney transplantation studies addressing model development, injury mechanisms, or interventions were included in the core synthesis, whereas unrelated studies or those lacking relevant experimental information were excluded. Conventional renal ischemia–reperfusion, isolated perfusion, and in vitro studies were not treated as direct transplantation evidence. Relevant reviews, clinical studies, and human molecular investigations were additionally considered for background and translational context.

## 2. Establishment of Rodent Kidney Transplantation Models

Although many rodent species are available for experimental research, kidney transplantation studies are currently performed mainly in rats and mice. These two model systems are technically mature, widely used, and valued for their relatively stable surgical workflows and genetic or immunological advantages. Both reproduce key aspects of clinical kidney transplantation, particularly vascular anastomosis and urinary tract reconstruction. However, because of the small size of experimental animals, these models require complex microsurgery. Their stability and reproducibility depend heavily on surgical skill, and intraoperative or postoperative complications can substantially affect animal survival, graft function, and interpretation of experimental results.

Vascular anastomosis is one of the critical determinants of success in rodent kidney transplantation. The anastomotic strategy affects not only surgical survival but also warm ischemia time, thrombosis risk, and early graft function, thereby influencing AKI and IRI outcomes. In mouse kidney transplantation, the most common approach is end-to-side anastomosis of the donor aortic segment to the recipient abdominal aorta, together with end-to-side anastomosis of the donor renal vein to the recipient inferior vena cava to restore graft perfusion [17]. Jin et al. described an orthotopic rat kidney transplantation method using sleeve arterial anastomosis and a modified renal venous stenting technique to improve the reliability of vascular reconstruction [10]. Gao et al. proposed a nonanastomotic rat orthotopic transplantation model to reduce the technical complexity associated with traditional vascular and ureteral reconstruction [18]. Yu et al. noted that conventional suturing in mouse kidney transplantation is prone to vascular thrombosis and therefore developed a dual cuff technique for both renal artery and renal vein anastomosis to reduce this risk [13].

Urinary tract reconstruction is another key factor affecting model stability. Failure of ureteral or bladder reconstruction may cause urinary leakage, ureteral necrosis, obstruction, and graft loss. Common approaches include anastomosis of the donor ureter–bladder patch to the recipient bladder or direct implantation of the donor ureter into the recipient bladder [9]. Wang et al. explored polyimide stent-assisted ureteral anastomosis in rat kidney transplantation to address limitations of traditional stent materials and placement techniques [19]. Preservation of ureteral blood supply is essential. Yin emphasized retaining the donor aortic patch and related branches to reduce ureteral necrosis and used 21G and 30G needles as guides for implantation of the donor ureter into the recipient bladder [14].

The greatest challenge of rodent kidney transplantation lies in the complexity of microsurgical procedures and the complications associated with them. Model stability directly determines the reliability of experimental findings, making standardization of established procedures essential. In practice, highly reproducible methodological papers and visualized protocols should be prioritized. Shrestha and Haylor described the surgical anatomy and microvascular techniques relevant to rat kidney transplantation and highlighted the importance of vascular anastomosis, urinary reconstruction, and perioperative management for model success, providing an important reference for surgical training and standardization [11,12,20]. You et al. presented a visualized rat orthotopic kidney transplantation model that can be used for training and reproducibility of experimental kidney transplantation procedures [21]. Because mouse kidney transplantation involves smaller vessels and ureters, it is even more dependent on standardized descriptions and training of key operative steps [14].

Native kidney management varies according to study objectives. Life-supporting models are widely used in mouse kidney transplantation and may involve bilateral native nephrectomy at the time of transplantation or delayed removal of the remaining native kidney 4–7 days after graft recovery. In contrast, non-life-supporting models retain the contralateral native kidney and are better suited for pathological and immunological studies [9]. In mechanistic studies of rodent transplant AKI, most life-supporting models ultimately remove both native kidneys so that the graft becomes the sole functioning kidney. Syngeneic or isogeneic transplantation is better suited for studying cold ischemia, warm ischemia, organ preservation, and IRI because it minimizes interference from alloimmune responses during early AKI. Allogeneic models are more appropriate for investigating the interaction between transplant AKI and rejection. Specialized models, including brain-dead donor, donation after circulatory death (DCD), and pediatric-to-adult kidney transplantation models, can simulate specific clinical scenarios. Esmaeilzadeh et al. established a brain-death induction model combined with rat kidney transplantation to examine the effects of brain-dead donors on transplant outcomes [22]. Pan et al. established a rat model in which juvenile donor kidneys were transplanted into adult recipients to study early compensatory graft growth [23]. These models increase the clinical relevance of rodent kidney transplantation by more closely reproducing distinct donor conditions and pathological contexts.

## 3. Rodent Models of Transplantation-Associated Acute Kidney Injury

**Figure 1:**
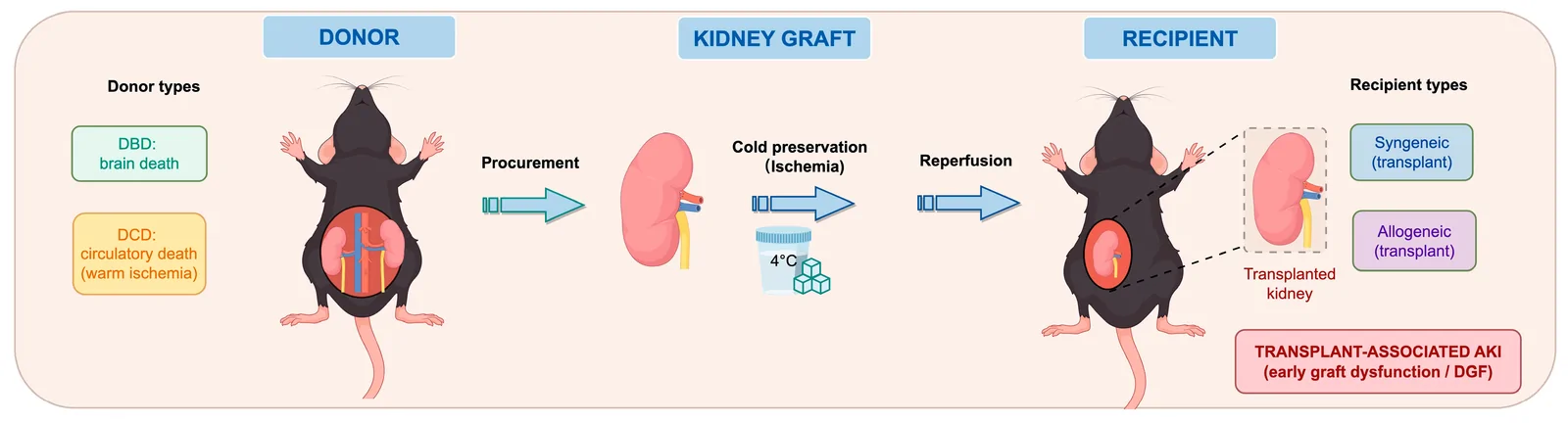
Experimental determinants of transplant-associated acute kidney injury in rodent kidney transplantation models.

Kidney transplantation-associated AKI is one of the principal applications of rodent kidney transplantation models. Unlike conventional renal IRI and isolated kidney perfusion systems, rodent kidney transplantation models incorporate the combined effects of organ procurement, preservation, transplantation surgery, reperfusion, and donor–recipient interactions, allowing more direct investigation of transplant-associated injury (Table 1). Cold ischemia, warm ischemia, and reperfusion are core variables used to establish transplant-associated AKI models. Cold ischemia mainly simulates donor kidney preservation, whereas warm ischemia may occur during donor procurement, DCD conditions, and vascular anastomosis. Reperfusion is the phase in which oxidative stress, endothelial injury, inflammatory cell infiltration, and cell death rapidly converge. Because reperfusion is an inherent component of kidney transplantation, it is generally not manipulated as an independent variable in conventional transplant models. Instead, investigators can establish AKI models with varying severity and pathological characteristics by manipulating cold and warm ischemia conditions in combination with donor characteristics and donor–recipient immunological settings (Figure 1).

Rodent kidney transplantation models reproduce major stages of clinical kidney transplantation, including donor organ procurement, ischemia, cold preservation, implantation, and reperfusion. Donor conditions can be modified to model donation after brain death (DBD) or donation after circulatory death (DCD), with DCD models involving a period of warm ischemia. Syngeneic transplantation minimizes the influence of alloimmune responses, whereas allogeneic transplantation enables investigation of the interaction between ischemia–reperfusion injury (IRI) and alloimmune responses. Following implantation and reperfusion, IRI contributes to transplant-associated acute kidney injury (AKI), which may be associated with delayed graft function (DGF).

Cold storage is a key experimental step that simulates donor kidney preservation and modulates the severity of transplant-associated AKI. By varying cold storage conditions, investigators can establish IRI models with distinct pathological features. Herrero et al. used a syngeneic rat kidney transplantation model to compare immediate transplantation with transplantation after 5 h of cold ischemia at 4 °C, thereby establishing IRI models under different cold ischemia conditions for evaluating graft injury and protective strategies [24]. Jain et al. used a mouse kidney transplantation model to compare cold ischemia alone, transplantation without cold ischemia, and transplantation following cold ischemia, providing experimental evidence for analyzing how preservation conditions shape post-transplant IRI [25]. Zhu et al. established syngeneic mouse kidney transplantation models with cold ischemia gradients ranging from 0.5 to 14 h and systematically assessed how cold ischemia duration affects graft injury severity [26].

Donor type is an important determinant of the clinical relevance of transplant AKI models. Donation after brain death (DBD) and DCD donor models have been incorporated into rodent kidney transplantation to mimic clinically relevant donor injury. Pratschke et al. established a brain-dead donor rat kidney transplantation model, thereby incorporating this clinical donor factor into experimental design for studying brain death-associated IRI [27]. Warm ischemia is another major determinant of IRI severity. Experimental protocols often vary warm ischemia duration or establish DCD models to reproduce different degrees of donor injury and assess their effects on graft recovery and subsequent damage. Kron et al. established a rat DCD kidney transplant model by inducing cardiac arrest through diaphragmatic incision, without prior heparinization or vascular clamping [28]. Wang et al. combined DCD rat kidney transplantation with different cold storage conditions to establish AKI models that more closely resemble marginal donor kidney transplantation in clinical practice [29].

The donor–recipient immunological setting is also an important variable in AKI model design. Syngeneic transplantation eliminates alloimmune responses as a confounding factor, whereas allogeneic transplantation more closely reflects the clinical transplant setting and enables investigation of the interaction between IRI and alloimmune responses. The choice should therefore be guided by the specific research question. Gueler et al. established an allogeneic mouse kidney transplantation model to investigate the combined effects of ischemic injury and alloimmunity [30]. Wang et al. established syngeneic and allogeneic DCD rat kidney transplantation models that simulated circulatory arrest-related warm ischemia, cold ischemia during preservation, and transplantation, providing experimental platforms for evaluating marginal donor-associated IRI and therapeutic strategies [31]. Accordingly, interpretation of rodent transplant AKI studies should take the immunological setting of the model into account.

Conventional non-transplant renal IRI models, commonly involving 30–45 min of renal pedicle clamping followed by reperfusion, provide a complementary approach for investigating ischemic kidney injury and recovery [32]. These models allow controlled assessment of renal functional recovery, tubular repair, and subsequent fibrosis, as well as evaluation of how interventions administered before or after ischemia influence injury severity and recovery trajectories [33]. However, because they do not reproduce the full transplantation process, protective effects observed in these models cannot be directly extrapolated to kidney transplantation.

Clinically, delayed graft function (DGF) is commonly defined as the need for dialysis within the first week after kidney transplantation [34]. In contrast, rodent transplantation studies generally assess early graft dysfunction using surrogate outcomes, including serum creatinine, blood urea nitrogen, histological injury, and graft survival. Additional biomarkers, such as kidney injury molecule-1 (KIM-1), neutrophil gelatinase-associated lipocalin (NGAL), and 4-hydroxynonenal (4-HNE), may provide complementary information on tubular injury and oxidative stress. However, these experimental endpoints are not equivalent to the clinical definition of DGF, and improvements in rodent graft outcomes should not be interpreted as evidence of established clinical efficacy.

## 4. Mechanistic Insights from Rodent Kidney Transplantation Models of AKI

**Figure 2:**
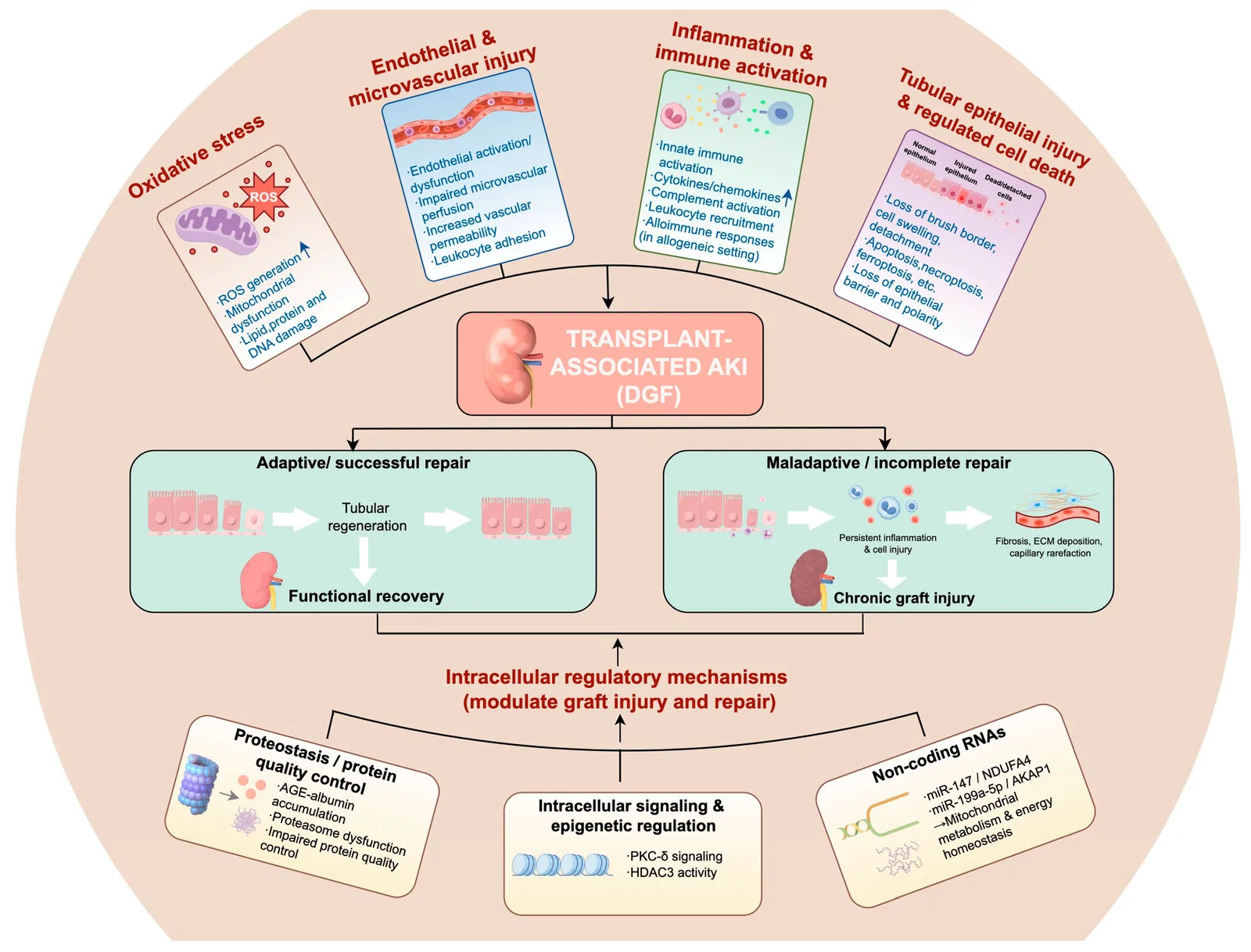
Mechanistic insights into transplant-associated AKI from rodent kidney transplantation models.

Rodent kidney transplantation models have expanded understanding of transplant-associated AKI from a simple ischemic insult to a dynamic process involving multiple interacting mechanisms. Current evidence indicates that oxidative stress, endothelial and microvascular injury, inflammatory and immune activation, cell death, and maladaptive repair jointly determine the onset, progression, and outcome of AKI (Figure 2).

Studies using rodent kidney transplantation models have implicated multiple mechanisms in transplant-associated acute kidney injury (AKI), including oxidative stress, endothelial and microvascular injury, inflammation and immune activation, and tubular epithelial injury and regulated cell death. Together, these pathological processes contribute to graft injury and early graft dysfunction. Subsequent graft outcomes are influenced by the balance between successful repair and persistent or maladaptive injury. Successful repair promotes restoration of tissue structure and function, whereas maladaptive repair is associated with persistent inflammation, fibrosis, microvascular loss, and chronic graft injury. Intracellular regulatory mechanisms involving proteostasis, protein kinase signaling, epigenetic regulation, and non-coding RNAs further modulate graft injury and repair. Abbreviations: AGE, advanced glycation end product; AKAP1, A-kinase anchoring protein 1; AKI, acute kidney injury; DGF, delayed graft function; DNA, deoxyribonucleic acid; ECM, extracellular matrix; HDAC3, histone deacetylase 3; miR, microRNA; NDUFA4, NADH dehydrogenase [ubiquinone] 1 alpha subcomplex subunit 4; PKC-δ, protein kinase C delta; RNA, ribonucleic acid; ROS, reactive oxygen species.

Oxidative stress is one of the earliest pathological processes in transplant-associated AKI. Reperfusion triggers rapid production of reactive oxygen species (ROS), resulting in lipid peroxidation, mitochondrial dysfunction, and cellular injury. Evidence from rodent kidney transplantation models further implicates oxidative stress and mitochondrial dysfunction in the pathogenesis of graft injury. Wagner et al. demonstrated that upregulation of heme oxygenase-1 (HO-1) attenuates IRI and improves graft survival [35], while Neto et al. reported improved graft function and reduced tissue injury following exogenous carbon monoxide (CO) treatment [36]. More recent studies have focused on mitochondrial ROS generation and direct ROS targeting. Lobb et al. demonstrated that mitochondria-targeted hydrogen sulfide donors preserve mitochondrial function and attenuate IRI in a rat kidney transplantation model [37], whereas Minami et al. showed that reducing excessive intragraft ROS, particularly H_2_O_2_, alleviates inflammation and improves graft outcomes in syngeneic rat kidney transplantation [38]. Collectively, these findings highlight the involvement of oxidative stress and mitochondrial dysfunction in transplant-associated AKI, with experimental evidence implicating HO-1/CO signaling, mitochondrial homeostasis, and intragraft ROS accumulation in the regulation of graft injury.

Endothelial and microvascular injury links early IRI to subsequent inflammatory amplification. Early IRI is characterized by dysregulated vascular tone and endothelial activation, followed by leukocyte adhesion, reduced microvascular perfusion, and sustained microvascular injury. Lin et al. showed that blockade of the thrombospondin-1/CD47 pathway restores nitric oxide-mediated protection, improves renal graft perfusion, and attenuates IRI [39]. Similarly, Dragun et al. demonstrated that ICAM-1 blockade reduces inflammatory cell recruitment [40], while Fuller et al. reported that inhibition of P-selectin-mediated leukocyte–endothelial interactions improves outer medullary microcirculatory perfusion and reduces tubular injury [41]. Together, these findings indicate that dysregulated vascular signaling and leukocyte adhesion are interconnected contributors to microvascular dysfunction and inflammatory amplification following transplantation. Beyond these early events, Herr et al. used dynamic contrast-enhanced MRI to demonstrate that prolonged cold ischemia is associated with sustained increases in renal graft capillary permeability and reductions in effective vascular volume [42]. These observations suggest that microvascular dysfunction may persist beyond the initial reperfusion period, although the extent to which these vascular alterations independently drive long-term graft injury remains uncertain.

Inflammation and immune activation are central mechanisms in transplant-associated AKI. Ischemia–reperfusion causes release of damage-associated molecular patterns (DAMPs), which activate innate immunity through pattern-recognition receptors such as Toll-like receptors (TLRs). In rodent kidney transplantation models, TLR4 deletion and TLR2 inhibition attenuated graft injury, supporting the involvement of TLR-mediated inflammatory signaling [43,44]. In addition, NF-κB inhibition has been shown to reduce monocyte infiltration [45], while carbon monoxide treatment suppresses proinflammatory cytokines, including IL-6, IL-1β, and TNF-α [36]. Together, these findings implicate interconnected TLR, NF-κB, and cytokine signaling pathways in early graft inflammation. Additional mechanisms include NLRP3 inflammasome activation associated with prolonged cold ischemia in DCD transplantation [29] and complement-mediated inflammation, as demonstrated by studies targeting C5a signaling and complement activation during preservation and reperfusion [46,47,48]. PD-1/PD-L1 interactions have also been implicated in regulating early T-cell infiltration after transplantation [49]. Although innate inflammatory signaling is supported by multiple experimental approaches, the relative contributions of inflammasome activation, individual complement pathways, and early T-cell responses remain less clearly defined across different transplantation settings.

Tubular epithelial injury and cell death form the direct histological basis of transplant AKI. Experimental evidence from rodent kidney transplantation models implicates both apoptosis and RIPK3-mediated necroptosis in graft injury. HO-1 induction has been associated with reduced tubular apoptosis and improved graft survival [35], whereas RIPK3-mediated necroptosis promotes HMGB1 release and amplifies post-transplant inflammation, contributing to graft injury and reduced survival [50]. Ferroptosis has emerged more recently, with miR-20a-5p attenuating cold ischemia-associated injury by targeting ACSL4-dependent ferroptosis [51]. A particularly important observation is that cold ischemia followed by transplantation induces tubular injury and cell death-related gene expression patterns distinct from those induced by cold ischemia alone or transplantation alone [25]. This suggests that transplant-associated AKI is not simply the sum of cold ischemia and reperfusion but has a distinct biological injury profile. Inconsistencies among cell death studies may therefore arise from differences in model design, cold ischemia duration, and the methods used to detect specific death pathways.

The significance of transplant AKI extends beyond early dysfunction; it can influence tissue repair and promote the development of chronic graft injury. Transcriptomic studies show that transplant injury activates shared injury–repair programs while retaining distinct molecular features, indicating heterogeneity in the graft response [52]. Zhu et al. further performed time-course RNA sequencing in a cold ischemia-associated mouse kidney transplantation model and found that apoptosis, DNA damage, and inflammatory genes dominate early after transplantation, whereas extracellular matrix remodeling, fibrosis, and metabolic reprogramming become prominent later [53]. Importantly, cold ischemia alone can induce late functional and structural injury even in the absence of alloimmunity [54], indicating that early IRI itself can drive chronic graft pathology. Wu et al. further implicated collectin-11 in tubulointerstitial fibrosis [55], while Yan et al. examined acute injury, inflammation, regeneration, and fibrosis within a syngeneic mouse kidney transplantation model [56]. Together, these studies support a link between early ischemic injury and subsequent tissue remodeling, although the mechanisms determining successful repair versus progressive fibrosis remain incompletely understood.

As understanding has deepened, research has expanded from classical pathophysiology to intracellular regulatory networks involving proteostasis, protein kinases, epigenetics, and non-coding RNAs. Cold ischemia-associated injury involves advanced glycation end product–albumin accumulation and proteasome dysfunction [57]. Zhu et al. demonstrated in a cold ischemia-associated mouse kidney transplantation model that protein kinase C-δ mediates tubular injury, while Xiang et al. showed that HDAC3 inhibition protects against cold ischemia/transplantation injury and improves graft function [26,58]. In addition, studies of miR-147/NDUFA4 and miR-199a-5p/AKAP1 suggest that microRNAs can influence cold ischemia-associated transplant AKI by regulating mitochondrial metabolism, energy homeostasis, and cellular injury responses [59,60]. These mechanisms are conceptually important but remain largely preclinical and are supported mostly by association or single-model intervention studies.

## 5. Interventions in Rodent Kidney Transplantation Models of AKI

Rodent kidney transplantation AKI models are used not only for mechanistic research but also for evaluating potential interventions. Transplant-associated AKI involves sequential pathological processes, including cold ischemia, ischemia–reperfusion, and immune activation. Because the dominant injury mechanisms differ across phases, interventional strategies are often stage-specific. According to timing, current approaches include donor pretreatment, organ preservation, reperfusion-targeted therapy, and postoperative recipient intervention (Table 2). These windows differ in translational implications. Preservation or machine perfusion interventions can act directly on the donor kidney while limiting systemic exposure, whereas perioperative or postoperative systemic treatment in recipients more closely resembles clinical pharmacotherapy but must be evaluated in the context of systemic safety and interactions with immunosuppression.

Donor pretreatment before organ procurement has been explored as a means of increasing graft tolerance to subsequent cold ischemia and reperfusion. Approaches include pharmacological pretreatment, ischemic conditioning, and other stress-conditioning strategies. Fuller et al. showed that donor pretreatment with N-acetylcysteine improves metabolic indicators after IRI in rat kidney grafts, without significant between-group improvements in creatinine or histological injury at 24 h [62]. Nelson et al. reported reduced apoptosis and lower acute tubular necrosis scores in a postoperative-day-3 subgroup after sodium thiosulfate donor pretreatment; however, recipient survival, renal function, and measured oxidative stress did not significantly improve [66]. Beyond pharmacological interventions, Redaelli et al. used heat shock preconditioning to induce donor HSP expression and improve function and survival of syngeneic rat renal isografts from non-heart-beating donors, demonstrating protection in this experimental setting [61]. Cicora et al. administered rabbit anti-rat thymocyte immunoglobulin to donors and showed that it attenuates syngeneic transplant IRI and promotes early graft recovery, suggesting that donor immune status can influence subsequent injury severity [65]. Li et al. found that donor melatonin pretreatment reduces transplant renal IRI by inhibiting NF-κB activation and apoptosis [64]. Gottmann et al. found that donor dopamine pretreatment improved post-transplant function, whereas dobutamine did not reproduce this benefit; catecholamine effects therefore should not be treated as interchangeable [63].

Organ preservation and perfusion are important targets for intervention in rodent models of transplant-associated AKI. Cold preservation can contribute to transplant-associated ischemia–reperfusion injury and early graft dysfunction. Traditional preservation strategies have focused on optimizing preservation solution composition and adding cytoprotective agents to reduce cold ischemic injury during ex vivo storage and improve tolerance to subsequent reperfusion. Schmitz et al. further showed that different combinations of flushing and storage solutions influence primary nonfunction, oxidative stress, and graft survival [70]. Protective additives such as sodium thiosulfate [76], the iron chelator deferoxamine [72], and estradiol [77] have been incorporated into preservation solutions and shown to reduce cold ischemia-associated injury and improve graft function to varying degrees. Another group of studies used gasotransmitters, bioactive molecules, and metabolic modulators with cytoprotective properties to increase graft tolerance to cold ischemia and reperfusion. Both CO and H_2_S have been shown to attenuate renal graft IRI [36,37]. Abe et al. reported that hydrogen-rich UW solution and high-pressure CO gas preservation reduce apoptosis and inflammation and improve graft function; hydrogen-rich UW solution significantly improved survival after 36 h, but not 24 h of cold ischemia [74,75]. Metabolic protectants such as propionyl-L-carnitine have likewise been shown to improve functional recovery after IRI and reduce early graft dysfunction [71,73]. Garcia-Cenador et al. found that cardiotrophin-1 treatment during organ preservation improves graft function and survival after prolonged cold ischemia, suggesting that cytoprotective cytokines may also be useful preservation-phase interventions [78]. Emerging non-pharmacological and targeted molecular strategies also show potential for promoting renal recovery. Hyperbaric oxygen therapy (HBOT) has shown renoprotective effects in experimental renal ischemia–reperfusion injury (IRI), potentially through modulation of oxidative stress, inflammation, and microvascular responses [86]. Limited transplantation-related evidence also suggests possible benefits in reducing early graft injury. Pharmacological inhibition of NADPH oxidases represents another approach to limiting oxidative injury, with preclinical renal IRI studies implicating NOX-dependent ROS generation in tubular damage [87]. However, evidence from conventional renal IRI models should not be directly extrapolated to kidney transplantation, and further validation in clinically relevant transplantation models is required.

Beyond improving overall graft tolerance, recent studies have attempted precise molecular modulation during the ex vivo phase to block IRI-related pathways before reperfusion. Vos et al. treated donor kidneys with a flush solution containing NF-κB decoy oligodeoxynucleotides before transplantation and reduced monocyte infiltration, indicating that inhibition of NF-κB-dependent inflammatory transcription during the ex vivo phase can be protective [45]. Lewis et al. added a C5a receptor antagonist to UW solution during cold storage and thereby blocked C5a receptor signaling, improving early graft survival and reducing inflammation and apoptosis [46]. Lin et al. flushed donor kidneys with UW solution containing an anti-CD47 monoclonal antibody during procurement and showed that blockade of thrombospondin-1/CD47 signaling improves renal graft perfusion and attenuates IRI, supporting microvascular protection during organ flushing [39]. Zheng et al. applied an siRNA cocktail during donor organ perfusion and preservation to downregulate IRI-related genes and reduce transplant injury, supporting further investigation of graft-directed nucleic acid delivery [81]. As molecular preservation strategies have evolved, new therapeutic targets continue to emerge. Hosszu et al. proposed the sigma-1 receptor as a novel target for improving renal cold ischemia injury [79]. Machine perfusion and new preservation modes have also attracted substantial attention. Kron et al. reported that hypothermic oxygenated perfusion (HOPE) improved DCD graft outcomes after 4 h but did not rescue grafts after 18 h of cold ischemia [28]. Subsequent work demonstrated immune modulation in an allogeneic series and in separate syngeneic DCD experiments; these were not a single allogeneic DCD protocol [82]. Abou Taka et al. evaluated a combination of 10 °C preservation, Hemopure, and sodium thiosulfate in a syngeneic orthotopic rat kidney transplantation model, but early biochemical improvements were not accompanied by significant recipient-survival or sustained renal-function benefit [80].

Reperfusion and recipient-directed therapies target the rapid inflammatory amplification and tissue injury that occur after blood flow is restored. Farrar et al. administered the anti-TLR2 antibody OPN301 to recipient mice before reperfusion in a kidney transplant IRI model and significantly improved graft function, suggesting that peri-reperfusion blockade of TLR2-mediated innate immune recognition is protective [44]. Yu et al. compared different complement-targeting approaches and showed that recipient anti-C5 treatment beginning before reperfusion attenuates injury; the effectiveness of alternative-pathway inhibition differed between preservation-phase and systemic administration [47]. Gueler et al. used EA-230 (AQGV), a synthetic anti-inflammatory tetrapeptide that mimics β-hCG activity, in an allogeneic mouse kidney transplantation model and found that it improves renal function, reduces tissue injury, and prolongs graft survival [30]. Beyond targeting inflammatory pathways, systemic cytoprotective treatment during the peri-reperfusion period has become an important area of investigation. Minami et al. developed the intragraft ROS-targeting polymer APP-103 and administered it intravenously 1 h before and 2 h after transplantation, resulting in improved renal function at days 1 and 7; the transplantation experiments did not establish a graft-survival benefit, and the 30-day observations came from a separate renal-clamping model [38]. Yan et al. administered synthetic bilirubin nanomedicine (BX-001N) early after transplantation and reported reduced early graft injury alongside early inflammation and day-7 fibrotic changes, illustrating the potential of multitarget cytoprotective strategies during the peri-reperfusion period [56]. Some interventions are not confined to a single therapeutic window. Cassis et al. compared darbepoetin alfa and carbamylated erythropoietin in rat kidney transplant IRI and found that consistent protection required combined donor pretreatment and recipient therapy [67]. Subsequent work further showed that xenon enhances IGF-1/IGF-1R-related repair responses, reduces cell death, T-cell infiltration, and fibrosis, and thereby delays cold ischemia-associated chronic allograft nephropathy [69]. Zhao et al. evaluated donor xenon pretreatment or early recipient xenon inhalation in rat allogeneic kidney transplantation and reported reduced early graft injury and inflammation following prolonged cold ischemia, as well as improved graft survival in combination with cyclosporine A [68].

Regenerative medicine strategies include cell therapy, extracellular vesicles, and pro-regenerative molecular interventions. Their effects involve immune modulation, attenuation of tissue injury, promotion of repair, and enhancement of renal regenerative capacity. Wu et al. showed that microvesicles derived from human Wharton’s jelly MSCs reduce IRI, inflammation, apoptosis, and fibrosis after DCD kidney transplantation, supporting further evaluation of a cell-free approach [84]. Cao et al. enhanced MSC homing by upregulating CXCR4 and thereby improved the protective effect of MSCs in a rat renal transplantation model [83]. More recently, regenerative repair strategies have expanded from cell therapy toward stimulation of endogenous repair. Yu et al. found that IL-13-associated signaling in the younger recipient setting attenuates post-transplant AKI and promotes renal regeneration through activation of JAK-STAT signaling in experiments comparing 6-week-old with 12-week-old recipients. IL-13 was given before transplantation and outcomes were assessed at 24 h; this does not establish treatment efficacy in aged recipients or after established AKI [85].

Although various interventions have shown protective effects in rodent kidney transplantation models, their interpretation requires consideration of experimental design and translational limitations. Many approaches have been evaluated in limited experimental settings, and independent replication across different transplantation models remains insufficiently established. Therapeutic timing is particularly important, as preventive donor or recipient treatment cannot be equated with intervention after established AKI. Preservation-phase strategies provide opportunities for graft-directed treatment, whereas systemic therapies require evaluation of adverse effects and interactions with immunosuppressive regimens. Although selected studies have examined longer-term outcomes or used allogeneic and DCD models, many reported benefits are based primarily on early functional or histological endpoints. Furthermore, findings from conventional renal IRI models should not be interpreted as direct evidence of efficacy in transplantation settings. Therefore, experimental protection should be regarded as preclinical evidence rather than established clinical efficacy or safety, and further validation across clinically relevant transplantation models with standardized outcomes and longer follow-up is needed.

## 6. Limitations and Future Directions

Although rodent kidney transplantation models are valuable for studying transplant-associated AKI, their establishment is highly dependent on microsurgical skill and lacks uniform reporting standards. Operator experience, vascular anastomosis, urinary reconstruction, and perioperative management can all affect surgical success and injury severity. Fuller et al. showed that donor kidney preservation mode and recipient treatment can influence functional outcomes [88]. Jain et al. compared tubular injury under different cold ischemia and reperfusion conditions and found that different injury phases produce distinct histological and molecular phenotypes, indicating that experimental designs should clearly distinguish cold ischemia from reperfusion injury [25]. Improving reproducibility and comparability requires standardized procedures of rodent kidney transplantation models. At minimum, reports should describe animal strain, donor–recipient pairing, syngeneic or allogeneic background, transplant approach, vascular and urinary reconstruction methods, cold and warm ischemia times, preservation conditions, DCD or DBD settings, recipient treatment, exclusion criteria, and functional and histological outcome measures. Anesthetic protocols represent an additional source of experimental variability. Isoflurane and ketamine/xylazine anesthesia may differentially affect systemic hemodynamics, renal perfusion, and inflammatory responses, while some anesthetics exhibit intrinsic cytoprotective effects in renal IRI models. These differences may confound comparisons of graft injury and recovery across studies and should therefore be considered when reporting and interpreting experimental outcomes [89,90].

Important gaps remain between rodent models and clinical kidney transplantation. Experimental animals are often young, healthy, and genetically homogeneous, whereas clinical donors and recipients frequently have advanced age, brain death, DCD status, diabetes, hypertension, infection, high immunological risk, or other comorbid conditions. Models combining DCD with cold storage better reproduce clinical DGF risk [28]. Studies indicate that aged kidneys are more prone to persistent inflammation and failed repair after AKI [91,92], supporting incorporation of aged donors or recipients into future transplant AKI models to improve clinical relevance. Pediatric-to-adult kidney transplantation models provide experimental platforms for studying transplantation of pediatric donor kidneys into adult recipients [23]. Vercauteren et al. showed that chronic renal failure and the uremic milieu can alter the severity of transplant AKI, indicating that recipient baseline status also influences model phenotype [93]. To reproduce the complex setting of highly sensitized patients, Qu et al. established a rat model of acute antibody-mediated rejection after kidney transplantation. This model showed how donor-specific antibody-induced complement activation and severe endothelial injury can jointly cause acute graft dysfunction and tubular necrosis, providing a platform for studying the influence of antibody-mediated immune injury on transplant-associated AKI [94]. Such composite models can improve the clinical relevance of rodent studies. Future models should better incorporate heterogeneity in donor quality and recipient background to more closely approximate clinical transplantation.

Intervention studies also face the problem of short-term efficacy with uncertain long-term benefit. Many studies use early postoperative serum creatinine, blood urea nitrogen, or histological injury as primary endpoints, but these measures do not necessarily predict long-term fibrosis, chronic graft dysfunction, or graft survival. A 24-week follow-up study in a syngeneic rat kidney transplantation model showed that early IRI has sustained effects on long-term graft outcomes [54]. Zhao et al. precisely controlled cold ischemia duration and followed grafts for up to 6 months, showing that early IRI can persistently affect long-term outcomes and promote chronic allograft nephropathy; graft function began to deteriorate around postoperative day 40 and clear chronic pathological changes were present by 6 months [69]. In a systematic review of 35 preclinical renal IRI studies, Lerink et al. found that approximately 68% (24/35) had follow-up shorter than 24 h, and 11 studies followed animals for less than 120 min. Such short observation windows are insufficient for evaluating post-injury repair and long-term graft outcomes [7]. Future intervention studies should therefore extend follow-up duration and combine multidimensional outcomes to assess repair and long-term consequences after acute injury. Publication bias may further complicate the interpretation of preclinical findings, as preferential reporting of positive results can lead to overestimation of experimental treatment effects. Although the extent of this bias in rodent kidney transplantation research remains uncertain, it highlights the importance of transparent reporting of both positive and negative findings. Together with the limited evidence on long-term outcomes, these concerns underscore the need for rigorous validation in clinically relevant transplantation models before experimental protective effects can be considered predictive of clinical efficacy [95].

With the development of multi-omics technologies, mechanistic research in transplant AKI is moving beyond conventional histology toward multidimensional molecular analysis. Traditional studies often rely on serum creatinine, blood urea nitrogen, and histological scoring, which cannot fully resolve dynamic changes across cell types and disease stages. Serkova et al. used 1H-NMR metabolomics to identify metabolic signatures of mild and severe IRI in rat kidney transplants, showing that metabolic profiles can reflect IRI severity and may help evaluate DGF risk [96]. Yu et al. combined transcriptomics, proteomics, and machine learning to analyze the effect of a young recipient milieu on rat kidney transplant AKI and identified key genes and cytokines related to immune regulation, providing a new approach for understanding complex regulatory networks [97]. In the future, integrating multi-omics data with histological findings, functional measures, and long-term outcomes, together with emerging approaches such as spatial omics, may improve the characterization of transplant-associated AKI and facilitate comparisons between experimental models and human disease.

Human molecular studies provide an additional perspective for evaluating the translational relevance of rodent kidney transplantation models. Using data from the Kidney Precision Medicine Project (KPMP) and other human kidney atlas initiatives, Lake et al. identified injury-associated epithelial states and altered tissue microenvironments [98], while Hinze et al. characterized epithelial stress responses in human AKI [99]. Although these studies provide evidence of human injury and repair responses, they primarily reflect native-kidney disease rather than transplantation-specific injury. Human allograft studies offer more direct evidence. Famulski et al. identified injury–repair transcriptional signatures associated with early graft dysfunction and recovery [100]. Cippà et al. demonstrated an early reperfusion-associated transcriptional response followed by divergent trajectories toward recovery or persistent injury and fibrosis, with early responses overlapping those observed in a mouse renal IRI model [101]. More recently, Pfefferkorn et al. identified corresponding injured epithelial cell states in mouse and human kidney allografts, with injury-associated signatures linked to poorer graft outcomes [102]. However, their study focused on T-cell-mediated rejection rather than peri-transplant IRI. Together, these findings suggest that epithelial stress, inflammatory responses, and maladaptive repair may represent shared features of experimental and human kidney injury. Nevertheless, molecular similarities do not establish identical causal mechanisms or predict therapeutic efficacy. Differences in injury context, donor characteristics, alloimmune responses, immunosuppression, and tissue sampling limit direct cross-species comparisons. Further validation in clinically relevant transplantation models and human allograft cohorts is therefore needed.

In addition, kidney transplant AKI should not be viewed solely as a local renal event. Zhao et al. showed in rats that renal graft IRI can trigger remote lung injury involving necroptosis and parthanatos-mediated cell death [103]. Subsequent studies suggested that kidney transplant IRI can contribute to remote liver injury through histone-mediated pyroptosis [104] and can aggravate remote lung injury through osteopontin-associated endoplasmic reticulum stress and necroptosis [105]. These findings indicate that future transplant AKI studies may need to expand their endpoints to include systemic inflammatory responses and remote organ injury in recipients, thereby providing a more comprehensive assessment of the systemic consequences of transplant-associated IRI.

## 7. Conclusions

Rodent kidney transplantation models provide an important bridge between basic mechanistic research and clinical prevention or treatment of transplant-associated AKI. Compared with conventional non-transplant renal IRI models, these models can reproduce sequential injury processes such as cold storage, warm ischemia, and reperfusion, while also incorporating clinically relevant factors including donor quality, recipient milieu, and alloimmunity. They are therefore particularly useful for studying mechanisms and potential interventions in transplant-associated AKI and DGF.

Nevertheless, rodent models cannot fully capture the complexity of clinical kidney transplantation. Their translational value depends on standardized model design, appropriate simulation of clinical scenarios, and continued improvement of outcome assessment. As organ preservation, precision immune modulation, and regenerative medicine continue to advance, rodent kidney transplantation models will remain indispensable for defining the dynamic mechanisms of transplant AKI, identifying new therapeutic targets, and supporting the translation of precision intervention strategies.

## Figures and Tables

**Table 1:** Comparison of experimental systems relevant to transplant-associated acute kidney injury.

Experimental System	Major Experimental Variables	Representative Outcome Measures	Key Limitations
Conventional renal pedicle-clamping IRI	Duration of warm ischemia and reperfusion; unilateral or bilateral clamping; systemic responses	Renal function, tubular injury, inflammation, and cell death	Does not reproduce organ procurement, cold preservation, vascular/urinary reconstruction, or donor–recipient interactions.
Isolated kidney perfusion	Perfusate composition, temperature, flow and pressure, oxygenation, and perfusion duration	Perfusion resistance, oxygen consumption, metabolic parameters, and injury biomarkers	Lacks an intact recipient and systemic immune responses; cannot independently establish post-transplant graft outcomes.
Syngeneic or isogeneic rodent kidney transplantation	Organ procurement, warm/cold ischemia, preservation, vascular and urinary reconstruction, and reperfusion	Graft function, histological injury, inflammation, survival, and long-term structural changes	Technically demanding; procedural variability affects reproducibility; does not model alloimmune mismatch.
Allogeneic rodent kidney transplantation	Transplantation-associated ischemic injury together with donor–recipient alloimmune mismatch; immunosuppression when used	Graft function, immune infiltration, rejection-related changes, survival, and chronic graft injury	Ischemic injury, alloimmunity, and immunosuppression-related effects may be difficult to disentangle.
Rodent kidney transplantation with additional donor- or preservation-related insults	Brain death or circulatory death, donor injury, prolonged cold ischemia, and preservation-related stress	Early graft dysfunction, histological injury, inflammation, survival, and longer-term graft outcomes	Greater complexity and variability; contributions of individual injury factors may be difficult to isolate.

Note: Donor- and preservation-related insults can be incorporated into syngeneic/isogeneic or allogeneic transplantation models; the categories are not mutually exclusive. Findings from conventional renal IRI or isolated kidney perfusion should not be interpreted as direct evidence of therapeutic efficacy after kidney transplantation. Abbreviations: IRI, ischemia–reperfusion injury.

**Table 2:** Interventions evaluated in rodent kidney transplantation models of AKI.

	Model	Intervention	Intervention Stage	Rationale	Reported Effects	Evidence Limitations
Redaelli et al., 2001 [61]	Syngeneic rat renal isografts after 20- or 40-min warm ischemia and 24 h cold ischemia	Donor hyperthermic preconditioning to induce heat-shock proteins	Donor pretreatment	Induce endogenous HSP-mediated stress tolerance before warm and cold ischemic injury	Improved renal isograft viability, function, and recipient survival	Seven-day follow-up in syngeneic grafts; donors recovered for 24 h after heat conditioning before procurement.
Fuller et al., 2004 [62]	Syngeneic rat kidney transplantation with 24 h cold ischemia	N-acetylcysteine	Donor pretreatment	Increase donor antioxidant capacity and glutathione-dependent defense before ischemia	Improved selected metabolic indicators (allantoin and trimethylamine-N-oxide); no significant improvement in creatinine or histological injury	Assessment at 24 h; metabolic changes were not accompanied by significant between-group improvements in creatinine or histological injury.
Gottmann et al., 2006 [63]	Syngeneic rat kidney transplantation with 24 h cold ischemia	Donor dopamine pretreatment, with other catecholamines as comparators	Donor pretreatment	Condition donor organs through neurohumoral cytoprotective effects beyond hemodynamic support	Dopamine improved post-transplant renal function; catecholamines were not uniformly protective	Transplant benefit was observed with dopamine, not dobutamine.
Li et al., 2009 [64]	Syngeneic rat kidney transplantation after 24 h cold ischemia	Melatonin (50 mg/kg by gavage) 2 h before donor nephrectomy	Donor pretreatment	Scavenge reactive oxygen/nitrogen species and inhibit NF-κB and apoptosis	Improved renal function and histology; increased SOD and reduced lipid peroxidation, iNOS, NF-κB, and caspase-3	Functional/histological endpoints at 6–24 h; mean survival increased from about 25 to 60 h, not durable rescue. Clinical dosing and safety remain unresolved.
Cicora et al., 2012 [65]	Syngeneic rat kidney transplantation after 3 h cold ischemia	Rabbit anti-rat thymocyte immunoglobulin, 12 h before procurement	Donor pretreatment	Modulate donor immune and inflammatory status before procurement	Improved early renal function and reduced necrosis, apoptosis, and proinflammatory mediators	Twenty-four-hour graft endpoints; absence of observed adverse infusion effects does not establish longer-term systemic safety.
Nelson et al., 2024 [66]	Syngeneic rat kidney transplantation with 24 h cold ischemia	Sodium thiosulfate	Donor pretreatment	Enhance antioxidant and hydrogen-sulfide-related cytoprotection before organ procurement	Reduced apoptosis and POD3 subgroup acute tubular necrosis scores; no significant renal-function or recipient-survival benefit	POD3 subgroup histological improvement; no significant benefit in recipient survival, renal function, or measured oxidative stress.
Cassis et al., 2011 [67]	Syngeneic and allogeneic rat kidney transplantation with 6–7.5 h cold ischemia	Darbepoetin alfa or carbamylated erythropoietin given to donors and recipients	Donor pretreatment/recipient treatment	Engage tissue-protective erythropoietin signaling while separating cytoprotection from erythropoiesis	Combined donor–recipient treatment was required for consistent functional and histological protection	Seven-day endpoints in syngeneic and allogeneic grafts; protection required donor plus recipient treatment, rather than recipient-only rescue.
Zhao et al., 2013 [68]	Allogeneic rat kidney transplantation after 24 h cold ischemia	Xenon inhalation for 2 h: donor treatment 24 h before retrieval or recipient treatment immediately after transplantation	Donor pretreatment/recipient treatment	Induce cytoprotective HSP-70 and HO-1 expression	Reduced early graft injury, inflammation, and early graft dysfunction and improved graft survival	Allogeneic model; the survival benefit was evaluated with cyclosporine A combination therapy and cannot be generalized to other immunosuppressive regimens.
Zhao et al., 2014 [69]	Allogeneic rat kidney transplantation after 16 h cold ischemia	Donor xenon pretreatment or early recipient xenon inhalation	Donor pretreatment/recipient treatment	Activate IGF-1/IGF-1R signaling to promote tubular repair	Reduced cell death, T-cell infiltration, and fibrosis and delayed chronic allograft nephropathy	Function assessed to day 40 and histology at 2 months in treated grafts; longer model-characterization follow-up should not be attributed to xenon treatment.
Vos et al., 2000 [45]	Allogeneic rat kidney transplantation with 30 min warm ischemia	NF-κB decoy oligodeoxynucleotides delivered in the graft-flush solution	Graft flushing	Inhibit NF-κB-dependent inflammatory transcription within the donor kidney before reperfusion	Reduced monocyte infiltration and intragraft inflammatory activation	Inflammatory endpoints at 24–72 h in nonimmunosuppressed allografts; these do not establish durable functional or survival benefit.
Lin et al., 2014 [39]	Syngeneic rat kidney transplantation with 6 h cold ischemia	Anti-CD47 monoclonal antibody added to UW graft-flush solution	Graft flushing	Block thrombospondin-1/CD47 signaling to preserve microvascular perfusion and nitric-oxide signaling	Improved graft perfusion and renal function and reduced tubular injury	Seven-day follow-up in syngeneic grafts; the survival experiment included five animals per group.
Schmitz et al., 2006 [70]	Syngeneic rat kidney transplantation after 16–24 h cold ischemia	HTK graft flush followed by UW storage, compared with single-solution protocols	Graft flushing/cold preservation	Optimize sequential flush and storage solutions to preserve energy metabolism and limit oxidative injury	The HTK/UW combination improved energy status and survival-related outcomes and reduced severe preservation injury	Survival followed for 28 days, but in syngeneic grafts; preservation-solution effects were dependent on cold ischemia duration and protocol.
Lobb et al., 2017 [37]	Allogeneic rat transplantation with NaSH during 24 h cold ischemia; separate syngeneic AP39 experiments	Hydrogen sulfide donors (NaSH or mitochondria-targeted AP39)	Graft flushing/cold preservation	Preserve mitochondrial function and bioenergetics during prolonged cold ischemia	Improved early graft function and survival and reduced oxidative stress, inflammation, and apoptosis	Fourteen-day follow-up; allogeneic NaSH and syngeneic AP39 experiments used different donors of H_2_S and should not be pooled as one protocol.
Mister et al., 2002 [71]	Syngeneic rat kidney transplantation with 4 h cold ischemia	Propionyl-L-carnitine	Cold preservation	Support mitochondrial fatty-acid metabolism and limit oxidative endothelial/tubular injury	Prevented deterioration of renal function and reduced structural and oxidative injury	Transplant function followed for 30 days; higher preservation concentrations (1.8 and 3.6 mg/mL) caused toxicity in pilot experiments.
Huang et al., 2003 [72]	Syngeneic rat kidney transplantation after 18 h cold ischemia	Deferoxamine added to preservation solution	Cold preservation	Chelate catalytic iron and limit hydroxyl-radical formation during cold ischemia and reperfusion	Improved renal function and reduced lipid peroxidation and tubular injury	Endpoints at days 3 and 9; the native contralateral kidney was retained until day 7 in the subacute experiment.
Azzollini et al., 2008 [73]	Allogeneic rat kidney transplantation with 6 h cold ischemia	Propionyl-L-carnitine	Cold preservation	Preserve mitochondrial energy metabolism and microvascular function during reperfusion	Improved early renal function (sCr) and 3-month survival; reduced tubular necrosis and inflammation	Ninety-day survival benefit was demonstrated with cyclosporine A; survival prolongation without immunosuppression was less pronounced.
Lewis et al., 2008 [46]	Syngeneic mouse kidney transplantation after 25–30 min warm ischemia and 30 min or 2 h cold ischemia	C5a receptor antagonist incorporated into UW preservation solution	Cold preservation	Inhibit complement-mediated inflammation during cold ischemia and early reperfusion	Improved early graft survival and reduced inflammation, apoptosis, and tissue injury	Mouse outcomes assessed at 72 h; accompanying human biopsy associations do not constitute therapeutic validation in patients.
Abe et al., 2012 [74]	Syngeneic rat kidney transplantation after 24–48 h cold ischemia	Hydrogen-rich UW preservation solution	Cold preservation	Scavenge cytotoxic radicals during cold ischemia and reperfusion	Improved graft function and reduced injury; survival improvement depended on cold-ischemia duration	Function and fibrosis followed to 90–100 days; survival improvement was significant after 36 h, but not 24 h, of cold ischemia.
Abe et al., 2017 [75]	Syngeneic rat kidney transplantation with 24 h cold ischemia	High-pressure carbon monoxide gas preservation	Cold preservation	Deliver carbon monoxide to the graft ex vivo to induce antiapoptotic and anti-inflammatory protection	Improved graft function and reduced apoptosis, inflammation, and tubular injury	Follow-up to 100 days; no significant survival difference among experimental groups; preservation required a specialized pressure chamber.
Garcia-Cenador et al., 2018 [76]	Syngeneic rat kidney transplantation after 24 h cold ischemia	Cardiotrophin-1	Cold preservation	Activate cytoprotective and prosurvival signaling during storage	Improved graft function and recipient survival and reduced tubular injury and apoptosis	Thirty-day follow-up; effective concentrations differed between addition at storage onset and delayed administration after 6 h.
Zhang et al., 2022 [77]	Syngeneic rat kidney transplantation after 24 h cold ischemia	Sodium thiosulfate-supplemented UW solution	Cold preservation	Augment antioxidant and cytoprotective capacity of UW solution during prolonged storage	Improved graft function and reduced oxidative stress, inflammation, apoptosis, and tubular injury	Fourteen-day follow-up in syngeneic grafts; storage supplementation and donor pretreatment are distinct protocols with different outcomes [59].
Zhou et al., 2022 [78]	Autologous rat kidney transplantation after 4 h cold ischemia	Estradiol	Cold preservation	Deliver estradiol during storage and assess local ERα/eNOS-associated protection	Lowered creatinine and BUN, reduced apoptosis and inflammation, and improved histopathology	Twenty-four-hour assessment in castrated male rats; unchanged circulating hormone levels are not a comprehensive systemic safety evaluation.
Hosszu et al., 2023 [79]	Rat autotransplantation with 2 h cold ischemia and 35 min warm ischemia; separate 24 h cold-storage experiments	Sigma-1 receptor activation with fluvoxamine during preservation	Cold preservation	Stabilize endoplasmic-reticulum/mitochondrial stress responses during cold ischemia	Improved graft function and reduced tubular injury, inflammation and apoptosis	Transplant follow-up was 24 h; 24 h cold-storage and receptor-knockout experiments provided nontransplant supporting evidence.
Abou Taka et al., 2024 [80]	Syngeneic orthotopic rat kidney transplantation with 24 h cold ischemia	10 °C preservation with Hemopure and sodium thiosulfate	Cold preservation	Combine subnormothermic storage, oxygen delivery, and antioxidant protection for marginal grafts	Selected early renal-function parameters improved; no significant recipient-survival or sustained functional benefit	Early biochemical signals were not accompanied by significant recipient-survival or sustained renal-function benefit.
Yu et al., 2016 [47]	Syngeneic rat kidney transplantation with 28 h cold ischemia	Preservation-phase complement inhibition or recipient anti-C5 treatment beginning before reperfusion	Cold preservation/recipient treatment	Interrupt complement activation during cold ischemia and reperfusion to prevent delayed graft function	Improved renal function and graft survival; reduced complement C3 deposition and apoptosis	Twenty-one-day follow-up; preservation-phase alternative-pathway inhibition was protective, whereas systemic delivery of the same inhibitor was not.
Zheng et al., 2016 [81]	Syngeneic mouse kidney transplantation with 4 h, 6 h or 12 h cold ischemia	Perfusion and preservation with an siRNA cocktail targeting IRI-related genes	Ex vivo perfusion/cold preservation	Silence multiple injury mediators within the isolated donor organ while limiting systemic exposure	Reduced inflammatory and apoptotic signaling and early graft dysfunction; prolonged survival after 6 h cold ischemia	Survival exceeded 80 days after 6 h storage; 12 h storage remained lethal despite delayed graft failure.
Kron et al., 2016 [28]	Syngeneic rat DCD kidney transplantation after 30 min warm ischemia and 4 h or 18 h cold ischemia	Hypothermic oxygenated perfusion (HOPE) before implantation	Ex vivo perfusion	Restore mitochondrial redox balance and reduce the reperfusion burst in marginal DCD grafts	Improved early function and survival after 4 h cold ischemia; did not rescue the 18 h cold-ischemia group	Seven-day follow-up; HOPE rescued grafts after 4 h of cold ischemia, but not after an otherwise lethal 18 h of cold ischemia; protection required oxygenation.
Kron et al., 2019 [82]	Allogeneic Lewis-to-Brown Norway transplantation; separate syngeneic DCD series with 30 min warm and 18 h cold ischemia	Hypothermic oxygenated perfusion (HOPE) before implantation	Ex vivo perfusion	Condition the graft and modulate innate and adaptive immune activation before reperfusion	Reduced inflammatory immune activation and improved graft function and survival	Ten-day follow-up; HOPE was compared with tacrolimus, not tested as its adjunct; the separate DCD series was syngeneic.
Neto et al., 2004 [36]	Syngeneic rat kidney transplantation with 24 h cold ischemia	Low-dose inhaled carbon monoxide	Recipient treatment	Activate cytoprotective signaling and limit oxidative and inflammatory reperfusion injury	Improved graft function and reduced tubular injury, apoptosis, and inflammation	Recipient carboxyhemoglobin increased during CO exposure; lack of evident acute toxicity does not establish human safety, despite prolonged survival.
Farrar et al., 2012 [44]	Syngeneic mouse kidney transplantation with 30 min cold ischemia	OPN301 anti-TLR2 antibody administered to recipients before reperfusion	Recipient treatment	Block TLR2-mediated innate immune recognition at reperfusion	Improved graft function and reduced inflammatory and tubular injury	Day-6 functional endpoint; the remaining native kidney was removed on day 5; no alloimmune mismatch.
Minami et al., 2020 [38]	Rat transplantation model characterized with 3, 6, or 12 h cold ischemia; APP-103 tested after 6 h	APP-103, an intra-graft ROS-targeting polymer, intravenously 1 h before and 2 h after transplantation	Recipient treatment	Scavenge reactive oxygen species selectively within the graft during reperfusion	Improved renal function at days 1/7; reduced oxidative stress, cytokines, apoptosis, and early fibrosis	Transplant endpoints at days 1/7; 30-day data came from clamping experiments.
Gueler et al., 2015 [30]	Allogeneic mouse kidney transplantation after 30 min warm ischemia and 1 h cold ischemia	EA-230 (AQGV), 1 h before and 6 h after surgery, then twice daily for 3 days	Recipient treatment	Suppress systemic and intragraft inflammatory amplification after transplantation	Improved renal function, reduced histologic injury, and prolonged graft survival	Four-week transplant follow-up; treatment started before surgery. Delayed treatment 24 h after AKI was tested in clamping, not transplantation.
Yan et al., 2026 [56]	Syngeneic mouse kidney transplantation after 24 min warm ischemia and 3.5 h cold ischemia	Synthetic bilirubin nanomedicine BX-001N administered early after transplantation	Recipient treatment	Provide antioxidant, anti-inflammatory, and antifibrotic protection during early graft injury	Reduced early AKI, inflammation, and day-7 fibrotic changes and showed early repair-associated changes	Days 1/7 endpoints in syngeneic grafts; day-7 fibrotic markers do not demonstrate durable antifibrotic efficacy.
Cao et al., 2013 [83]	Allogeneic rat kidney transplantation with 1 h cold ischemia	CXCR4-upregulated MSCs administered 24 h after transplantation	Regenerative repair/recipient treatment	Enhance MSC homing to the injured graft and strengthen paracrine repair and immunomodulation	Improved graft function and histology and reduced inflammation and apoptosis	Day-3 endpoints with genetically modified cells; authors identified small sample size and lack of long-term survival/tolerance assessment.
Wu et al., 2018 [84]	Rat DCD kidney transplantation (Sprague–Dawley donors/recipients) with 50 min warm ischemia and 18 h cold ischemia	Microvesicles derived from human Wharton’s jelly mesenchymal stromal cells	Regenerative repair/recipient treatment	Deliver paracrine regenerative and immunomodulatory cargo without whole-cell administration	Improved renal function and reduced inflammation, apoptosis, oxidative injury, and fibrosis	Fourteen-day follow-up; human vesicles were administered to rats, leaving cross-species product translation and durable benefit unresolved.
Yu et al., 2024 [85]	Allogeneic rat kidney transplantation comparing 6-week-old and 12-week-old recipients	Recipient IL-13 supplementation on day −1 and immediately before transplantation	Regenerative approach/recipient pretreatment	Recreate a youthful pro-regenerative systemic environment around transplantation	Reduced AKI and apoptosis and promoted tubular regeneration and early functional improvement	Twenty-four-hour endpoints; recipients were 6 versus 12 weeks old, not aged animals. Preventive IL-13 dosing does not establish post-AKI rescue or durable benefit.

Note: Intervention stages were classified according to the timing of treatment relative to organ procurement, preservation, reperfusion, and transplantation. Reported protective effects reflect the findings of the original experimental studies and should not be interpreted as evidence of comparative efficacy across interventions or established clinical efficacy or safety. Evidence limitations summarize selected study-specific constraints related to treatment timing, follow-up duration, experimental models, and safety assessment. The absence of reported adverse effects should not be interpreted as evidence of safety. Numbers in square brackets correspond to references in the main text. Abbreviations: AKI, acute kidney injury; BUN, blood urea nitrogen; C5a, complement component 5a; CD47, cluster of differentiation 47; CXCR4, C-X-C chemokine receptor type 4; DCD, donation after circulatory death; eNOS, endothelial nitric oxide synthase; ERα, estrogen receptor alpha; HOPE, hypothermic oxygenated perfusion; HSP, heat-shock protein; HTK, histidine–tryptophan–ketoglutarate; IGF-1/IGF-1R, insulin-like growth factor 1 and its receptor; IL-13, interleukin 13; iNOS, inducible nitric oxide synthase; IRI, ischemia–reperfusion injury; MSC, mesenchymal stromal cell; NF-κB, nuclear factor kappa B; ROS, reactive oxygen species; siRNA, small interfering RNA; SOD, superoxide dismutase; TLR2, Toll-like receptor 2; UW, University of Wisconsin; CO, carbon monoxide; H_2_S, hydrogen sulfide; NaSH, sodium hydrosulfide; POD, postoperative day.

## Data Availability

No new data were created or analyzed in this study. Data sharing is not applicable to this article.
